# Diagnostic significance of CD10 marker to differentiate colorectal adenocarcinoma from adenomatous polyp: A pathological correlation

**DOI:** 10.22088/cjim.15.2.228

**Published:** 2024

**Authors:** Ghasem Dehini, Hossein Ghorbani, Soraya Khafri, Javad Shokri Shirvani, Akramossadat Hosseini, Sahar Sadr Mohararpur, Tina Rouhi

**Affiliations:** 1 Clinical Research Development Unit of Rouhani Hospital, Babol University of Medical Sciences, Babol, Iran; 2 Department of Pathology, Faculty of Medicine, Babol University of Medical Sciences, Babol, Iran; 3 Social Determinants of Health Research Center, Health Research Institute, Babol University of Medical. Sciences, Babol, Iran; 4 Department of Gastroenterology, Faculty of Medicine, Babol University of Medical Sciences, Babol, Iran; 5 Non-Communicable Pediatric Diseases Research Center, Health Research Institute, Babol University of Medical Sciences, Babol, Iran

**Keywords:** Immunohistochemistry, Colorectal neoplasms, Neprilysin, Adenomatous polyps

## Abstract

**Background:**

Colorectal cancer could be developed from adenomatous polyp. The study aimed to evaluate the diagnostic significance of stromal and epithelial CD10 (Neprilysin) expression in patients with colorectal adenocarcinoma and adenomatous polyps.

**Methods:**

This cross-sectional study was conducted on 141 patients with colorectal adenocarcinoma and adenomatous polyps referred to Ayatollah Rouhani Hospital from March 2020 to March 2021. Differential diagnoses of colorectal adenocarcinoma and adenomatous polyps were made colonoscopically, and then samples were taken from the lesions. The pathologists confirmed the final diagnosis as colorectal adenocarcinoma, high-grade or low-grade adenomatous polyps. The stromal and epithelial CD10 expression was evaluated by immunohistochemistry. The data was analyzed by SPSS 22 software (p<0.05).

**Results:**

Sixty-five (46.1%) of the cases were low-grade polyps that were included positive (4 cases; 6.20%) and negative (61 cases; 93.80%) CD10 expression (P=0.001), also 76 (53.9%) of them were either high-grade polyps (21 cases) or adenocarcinomas (21 cases). Also, epithelial CD10 expression was significantly higher in the well-differentiated adenocarcinoma (38 cases) group than moderate (13 cases) and poor (4 cases) groups (P =0.001). Moreover, the CD10 expression level in the adenomatous polyps (10 positive cases and 76 negative cases) was correlated with the degree of dysplasia (P = 0.001) and the presence of tumor invasion (8 positive cases and 133 negative cases) (P = 0.001).

**Conclusion:**

The CD10 expression is associated with an increased degree of dysplasia and the presence of tumor invasion in patients with pre-neoplastic lesions and colorectal adenocarcinoma.

Colorectal cancer (CRC) is known as the fourth and the third most common cancer in males and females, respectively, which is causing cancer-related mortality worldwide (1). Approximately, 1.8 million new CRC cases and 880,792 deaths have been reported in 2018, based on World Health Organization (2), and the GLOBOCAN database (2). The incidence pattern of CRC is varied regionally (3), depending on lifestyle, genetic, epigenetic, and environmental factors (4). The five-year prevalence of CRC has been estimated at 29.7 per 100.000 for the Iranian population in 2018 (5). Colorectal cancer is currently classified into different subtypes, which are categorized by differentiation degree (histological grade), molecular alterations, and tumor stage (6). One of the most common types of colorectal cancer is colorectal adenocarcinoma and it is considered the second cause of cancer-related mortality (7). 

Hence, regular screening is important to find colorectal polyps, detect cancerous and pre-cancerous lesions, and increase the survival rate (10). Endoscopy (including sigmoidoscopy and colonoscopy) is a gold standard tool for the diagnosis of precancerous lesions and colorectal neoplasms, which also allows for obtaining 3D images and tissue samples for histological assessment (11, 12). Despite the many advances in imaging techniques, the early detection of colorectal cancer and adenomatous lesions is still a major challenge (12). Currently, molecular markers such as protein expression are used to evaluate the diagnosis, progression of CRC, and even response to therapy (13). CD10, also known as neprilysin or Common Acute Lymphoblastic Leukemia Antigen (CALLA), is a cell-surface zinc-dependent metalloprotease. It is physiologically expressed by a variety of cells, including neutrophils, B cells, the epithelial cells of the small intestine, kidney, breast, lung, prostate, and some lymphoid progenitor cells (14). The overexpression of CD10 has been reported in epithelial and stromal malignant cells of bladder tumors, and cervical, breast, lung, renal, and liver cancers. It is also related to clinical outcomes, metastasis, tumor stage, and therapeutic resistance (15-22). CD10 can play a key role in tumorigenesis by interaction with signaling pathways (23). Some studies have also shown that the expression of CD10 is enhanced in colorectal cancer and related to infiltrating inflammatory cells, liver metastasis, higher grade of tumor, and poor prognosis (24-27). However, its diagnostic significance has not been proven in colorectal adenocarcinoma and adenomatous polyps. This present study aimed to evaluate the potential use of CD10 as a differential diagnostic marker between colorectal adenocarcinoma and adenomatous polyps and between two grades of dysplasia in adenomatous polyps.

## Methods

This cross-sectional study was conducted at the Pathology Department of Ayatollah Rouhani Hospital, Babol from March 2020 to March 2021 and approved by the Medical Research Ethics Committee of Babol University of Medical Sciences (Ethics no: IR.MUBABOL.HRI.REC.1398.002). The subjects of this study were 141 patients initially diagnosed by colonoscopy and underwent biopsy at the same time. We excluded subjects with malignancy other than colon cancer or patients who underwent chemotherapy and surgery. Ten adjacent normal colorectal tissue specimens are used as the negative control samples. Formalin-fixed, paraffin-embedded tissue blocks were prepared from them. The diagnosis of adenocarcinoma or adenomatous polyps was made by two independent pathologists based on histopathological examination of Hematoxylin and Eosin (H&E) -stained sections. Then categorized into; (1) “well-differentiated adenocarcinoma”, (2) “moderately differentiated adenocarcinoma”, (3) “poorly differentiated adenocarcinoma”, (4) “low-grade adenomatous polyps”, and (5) “high-grade adenomatous polyps”. 

We evaluated CD10 expression in specimens using an immunohistochemical staining method. One block from each sample was selected for testing with anti-CD10 markers (Dako, Japan). Sections were deparaffinized in xylene overnight and rehydrated in graded alcohol series. Antigen retrieved in citrate buffer (pH = 9.0). 

**Figure 1:**
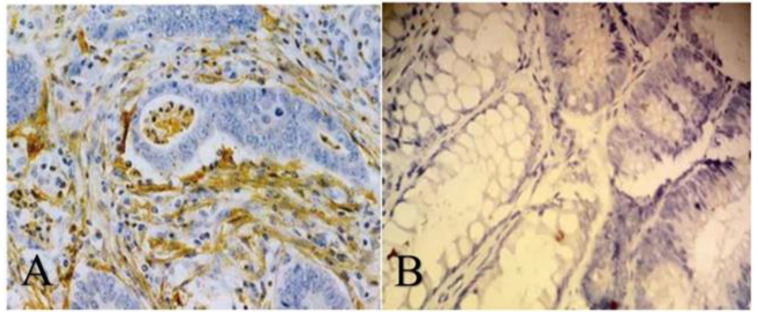
A. Stromal CD10 expression is observed along invasive tubules of colorectal adenocarcinoma (CD10 Immunostaining, Magnification 100X). B. CD10 expression is negative along neoplastic tubules in low-grade adenomatous polyps (CD10 Immunostaining, Magnification 400X)

Finally, primary and secondary antibodies were added for staining according to the provided protocol. Normal endometrium samples were used as a positive control. CD10 expression was considered positive when more than or equal to 10% of stromal and/or epithelial (neoplastic) cells were positive for CD10 with the membranous and or cytoplasmic pattern of staining (figure 1). 

Data analysis was carried out by SPSS 22 (SPSS Inc., Chicago, Ill., USA). Frequency rate and, frequency percentages were calculated as variables. The chi-square test was used to compare groups and evaluate the relationships between clinicopathologic features and stromal/epithelial CD10 expression (p < 0.05). 

## Results

A total of 141 cases of colorectal adenocarcinoma and adenomatous polyps were included. Eighty-six (61%) samples were adenomatous polyps and fifty-five (39%) were adenocarcinoma (table 1). In general, CD10 positivity rates were 33.3% and 5.7% in stromal and epithelial tumor cells, respectively. Stromal CD10 positive staining was detected in adenocarcinoma more than in adenomatous polyps (P = 0.001). The evaluation of CD10 in epithelial neoplastic cells reveals only 14.6% positivity in adenocarcinoma specimens and none in adenomatous polyps. According to statistical analysis, the epithelial CD10 positivity rate was significantly different between the adenocarcinoma group and the adenomatous polyps group (P = 0.001) (table 2). We compare stromal/ epithelial CD10 positivity rates between the adenocarcinoma group and high-grade adenomatous polyps. Our finding indicates that stromal CD10 (sCD10) expression in the adenocarcinoma group was higher than high-grade adenomatous polyps (67.3% vs. 28.6%, P = 0.002), while no significant difference in epithelial CD10 (eCD10) expression was observed between two groups (P = 0.06) (table 3). 

**Table 1:** Colorectal adenocarcinoma and adenomatous polyp’s distribution

**Variables**	**Total**
**Adenocarcinoma differentiation**	N (%)
**Well**	38 (27)
**Moderate**	13 (19.2)
**Poor**	4 (2.8)
**Adenomatous polyps**	N (%)
**Low grade**	65 (46.1)
**High grade**	21(14.9)
**Total**	141 (100)

**Table 2:** Stromal/epithelial CD10 in colorectal adenocarcinoma and adenomatous polyps

**Variables**	**No. of cases**	**Stromal CD 10** **N (%)**		**P-value**	**Epithelial CD10** **N (%)**		**P-value**
		**Positive**	**Negative**		**Positive**	**Negative**	
Adenocarcinoma	55	37 (67.3)	18 (32.7)	0.001*	8 (14.6)	47 (85.4)	0.001*
Adenomatous polyps	86	10 (11.6)	76 (88.4)		0 (0)	86 (100)	

*P ˂0.05, Chi-squared test

**Table 3:** CD10 expression in colorectal adenocarcinoma and high-grade adenomatous polyps

**Variables**	**No. of cases**	**Stromal CD 10** **N (%)**		**P-value**	**Epithelial CD10** **N (%)**		**P-value**
		**Positive**	**Negative**		**Positive**	**Negative**	
Adenocarcinoma	55	37 (67.3)	18 (32.7)	0.002*	8 (14.6)	47 (85.4)	0.06
High-grade polyps	21	6 (28.6)	15 (71.4)		0 (0)	21 (100)	

*P ˂0.05, Chi-squared test

Stromal CD10 staining was detected more in moderately differentiated adenocarcinoma compared to well or poorly-differentiated adenocarcinoma (P = 0.001). Also, epithelial CD10 positivity rates were higher in well-differentiated adenocarcinoma among different groups of adenocarcinomas (21.1% versus 0%, P = 0.17) (table 4). According to table 5, stromal CD10 was positive in 28.6% (6/21) of high-grade and 6.2% (4/65) of low-grade polyps. There is a significant difference in stromal CD10 expression between high and low-grade polyps (P = 0.001). All of the epithelial cells in adenomatous polyps were negative for CD10 marker (table 5).

**Table 4:** CD10 expression in adenocarcinoma groups

**Variables**	**No. of cases**	**Stromal CD 10** **N (%)**		**P-value**	**Epithelial CD10** **N (%)**		**P-value**
		**Positive**	**Negative**		**Positive**	**Negative**	
Well	38	26 (68.4)	12 (31.6)	0.001*	8 (21.1)	30 (78.9)	0.17
Moderate	13	9 (69.2)	4 (30.8)		0 (0)	13 (100)	
Poor	4	2 (50)	2 (50)		0 (0)	4 (100)	

*P ˂0.05, Chi-squared test

**Table 5:** CD10 expression in high-grade and low-grade polyps

**Variables**	**No. of cases**	**Stromal CD 10** **N (%)**		**P-value**	**Epithelial CD10** **N (%)**		**P-value**
		**Positive**	**Negative**		**Positive**	**Negative**	
**High-grade polyps**	21	6 (28.6)	15 (71.4)	0.001*	0 (0)	21 (100)	-
**Low-grade polyps**	65	4 (6.20)	61 (93.80)		0 (0)	65 (100)	

*P ˂0.05, Chi-squared test

## Discussion

Colorectal adenocarcinoma is among the common neoplasms worldwide. Early diagnosis of colorectal adenocarcinoma is a key factor in clinical outcome and prognosis (28). Some biological markers such as CD10 may help in the diagnosis of precancerous lesions and colorectal malignancies in the early stages (25, 29). The current study has examined the CD10 expression in differentiation between colorectal cancer and adenomatous polyps. In this study, CD10 was detected in stromal cells of 47 adenocarcinomas (78.9%) and epithelial cells of eight (14.6 %) with the same. The presence of CD10 was higher in stromal cells than epithelial cells. Also, the CD10 expression is significantly higher in colorectal adenocarcinoma than adenomatous polyps (81.9% vs. 11.6%). These findings suggest that the CD10 marker may be effective in distinguishing colorectal adenocarcinoma from adenomatous polyps. Similarly, Abinaya et al. showed a gradually significant increase in CD10 expression in low-grade adenomas to high-grade adenomas and the highest in adenocarcinomas (30).

In contrast, Magadhi et al. indicated that no significant difference was found between adenomas and adenocarcinomas in stromal CD10 positivity (24). Moreover, other studies were conducted to evaluate the diagnostic role of CD10 markers in categorizing some lesions. Ahuja et al. studied that CD10 expression was raised in hepatocellular carcinoma compared to metastatic cancer, which was useful in differentiating these two lesions from each other. Increased CD10 expression has been reported in breast cancer, malignant thyroid lesions, papillary thyroid carcinoma, and malignant melanoma (31-33). 

Based on the above findings, it seems that CD10 expression can be used as an additional tool for early diagnosis, and response to therapy. In our study, stromal cells showed considerable expression of CD10, especially in well and moderately-differentiated adenocarcinomas (68.4% and 69.2% respectively). Stromal expression of CD10 was 50% in poorly differentiated adenocarcinoma, 28.6%, and 6.2% in high and low-grade adenomatous polyps. Although the highest expression of CD10 in epithelial cells was seen in well-differentiated adenocarcinoma, it was not statistically significant. These findings suggest that higher expression of CD10 may be related to the degree of cell differentiation. Stromal CD10 expression in high-grade polyps was significantly higher than in low-grade adenomatous polyps, which was similar to the Ogawa et al.’s study (25). 

Thus, the presence of stromal CD10 can be relevant to severe dysplasia. Data from another study confirmed our results. It reported a significant difference in CD10 immunohistochemical expression between colorectal adenocarcinoma and adenomas. They showed that tumor CD10 expression gradually increased from 20% in low-grade to 50% in high-grade adenomas, and reached 80% in invasive colorectal adenocarcinoma cases (34). Żurawski et al. showed significant positive correlations between serum and tissue levels of CD10 with colorectal cancer stages in 113 patients (35). CD10 or neprilysin is a cell surface protein that is expressed in early lymphoid progenitor stages and involved in the pathogenesis of several cancers. Probably, the role or mechanism of CD10 in the pathogenesis of colorectal cancer is induced by interaction with adhesion kinase, PI3K-AKT, and other signaling pathways, promoting cell proliferation and motility, inhibiting apoptosis, up-regulating the matrix metalloproteinase (MMPs), and promoting angiogenesis and tumorigenesis (36, 37). Also, it seems that the development and progression of colorectal carcinoma correlated to increased levels of CD10 and P53 along with decreased MUC2 expression (37, 38). On the other hand, a study reported that CD10 antibodies can inhibit cell proliferation by activating cyclin-dependent kinase pathway in the animal model of malignant mesotheliomas. They also indicated that aggressive histological types were related to tumoral CD10 expression. It seems that CD10 mAb can be chosen as an effective target for treatment of malignant mesotheliomas (39). Therefore, expression analysis of CD10 along with other diagnostic tools may be helpful in early diagnosis, favorable response to cancer treatment, therapeutic target, prognosis, and patient survival. Results of one recent study have shown that CD10 expression was significantly lower in mucinous and signet ring carcinoma than adenomas (40). This study had several limitations. First, we did not investigate CD10 expression according to tumor size, metastasis, histological patterns, and clinical stages. Second, we also did not examine the relationship between CD10 expression and cancer recurrence. Future research should consider these points. The current study suggests that overexpression of CD10 should be considered a diagnostic factor in differentiating colorectal cancer from adenomatous polyp. According to our data, the CD10 expression is related to an increased degree of dysplasia and the presence of invasion in patients with colorectal adenocarcinoma and preneoplastic lesions. However, more studies are warranted to evaluate the relationship between CD10 and tumor size, metastasis, cancer recurrence, histological patterns, and clinical stages in larger groups of patients.
